# A four-part working bibliography of neuroethics: part 1: overview and reviews – defining and describing the field and its practices

**DOI:** 10.1186/1747-5341-9-9

**Published:** 2014-05-16

**Authors:** Liana Buniak, Martina Darragh, James Giordano

**Affiliations:** 1 Neuroethics Studies Program, Edmund D. Pellegrino Center for Clinical Bioethics, Georgetown University Medical Center, Washington, DC 20057, USA; 2 Bioethics Research Library, Kennedy Institute of Ethics, Georgetown University, Washington, DC 20057, USA; 3 Inter-disciplinary Program in Neuroscience and Department of Neurology, Georgetown University Medical Center, Washington, DC 20057, USA; 4 Human Science Center, Ludwig-Maximilians Universität, München, Germany

**Keywords:** Neuroethics, Neuroscience, Neurobioethics, Bioethics, Ethics, Bibliography

## Abstract

**Background:**

Neuroethics entails investigations of neurocognitive mechanisms of morality and ethics; and studies and address of the ethical issues spawned by the use of neuroscience and its technologies to investigate cognition, emotion and actions. These two principal emphases, or what have been called “traditions” of neuroethics both mirror traditional bioethical discussions (such as debates about the safety of technological and pharmaceutical advances and ethical implications of new scientific and technological discoveries), and engage discourse about neuroscientific investigations of (proto-moral and moral) cognition, emotions and behaviors, and what such findings may mean for human beliefs and conduct - from the individual to the political levels.

Given the growth, range, and rapid maturation of the field of neuroethics we provide an iterative, four-part document that affords a repository of international papers, books, and chapters that address the field in overview, and present discussion(s) of more particular aspects and topics of neuroethics. This first installment lists reviews and overviews of the discipline, and broad summaries of basic developments and issues of the field.

**Methods:**

To systematically survey the neuroethics literature, searches were performed by accessing 11 databases, 8 additional literature depositories, and 4 individual journal searches using indexing language for National Library of Medicine (NLM) Medical Subject Heading databases. Searches and assurance against overlapping coverage were conducted using the *RefWorks* citation management program.

**Results:**

Overview, review and reflections upon the history and multicultural perspectives of neuroethics were obtained and relevant listings from international journals, books, and book chapters are provided. Part I will be followed by three installments that will address a): the neuroscience of morality and ethics, including discussions of free will, and personal autonomy; b) “second tradition neuroethics”, to include specific ethical issues in neuroscience; clinical neuroethics; and c) neuroethics education/training; neuroethics and society; neuroethics and law; neuroethics and policy; and international neuroethics.

## Introduction and background

Neuroscience has employed and built upon an existing body of research from the natural, physical and social sciences, as well as the humanities in attempts to establish a comprehensive understanding of the structure and function of nervous systems and the brain. Utilizing ever more sophisticated tools, the multi-disciplinary approaches of neuroscience have enabled a number of exciting discoveries, and concomitantly challenged extant ideas about the relationship of brain and mind, and what such constructs—as well as the direction and momentum of neuroscientific inquiry itself—might mean and incur for the philosophies, moral beliefs, attitudes and values, ethical standpoints, and laws that define the social sphere.

Addressing such difficult questions—and answers—is the basis of the discipline of neuroethics. The term, “neuroethics” was introduced by Anneliese Pontius in a paper entitled “Neuro-ethics of ‘walking’ in the newborn”, which appeared in August 1973 in the journal *Perceptual and Motor Skills*[1]. The concept of “a neuroethics” (to include both the “neuroscience of ethics”, and the “ethics of neuroscience”) was defined and advanced by Adina Roskies [2] and the term – and field - became broadly, if not publicly identified as a result of William Safire’s opening lecture “Visions for a New Field of ‘Neuroethics’” at the 2002 Dana Foundation conference *Neuroethics: Mapping the Field*[3]. Considered to be the discipline’s “coming out conference” speakers called attention to neuroethical areas of inquiry, which encompass “…what is right and wrong, good and bad about the treatment of, or unwelcome invasion of and worrisome manipulation of the human brain” [4]. Yet, such a definition belied the richness of this newly emerging field.

Re-appropriating Roskies’ definition, neuroethics entails investigations of neurocognitive mechanisms of morality and ethics; and studies and address of the ethical issues spawned by the use of neuroscience and its technologies to investigate cognition, emotion and actions. These two principal emphases, or what have been called “traditions” of neuroethics both mirror traditional bioethical discussions (such as debates about the safety of technological and pharmaceutical advances and ethical implications of new scientific and technological discoveries) and directly engage neuroscientific investigations of (proto-moral and moral) cognition, emotions and behaviors, and philosophical, ethical and legal reflections upon what such findings may mean for human beliefs and conduct - from the individual to the political levels.

As a consequence of deepened interest and investments in the neurosciences, including for example, the United States’ (US) congressionally-declared Decade of the Brain (1990–1999), Decade of Pain Control and Research (2000–2009), the newly declared *Brain Research through Advancing Innovative Neurotechnologies* (BRAIN) initiative (http://www.nih.gov/science/brain/), and a number of international programs (such as the European Union’s *Human Brain Project,* and the Asian *Decade of the Mind*), the importance of neuroethics – as a set of practices and a discipline – increased, and research centers specifically dedicated to advancing neuroethics were established. Professional societies such as the Society of Neuroscience (SfN) and the International Brain Research Organization (IBRO) further encouraged open discourse regarding implications of neuroscience research and its social utility. The Dana Foundation (US), the United Nations Educational, Scientific, and Cultural Organization (UNESCO), World Health Organization (WHO), and the International Neuroethics Society (INS) have all been important to opening international communication in and about neuroethics by encouraging the “cross-fertilization of ideas” at annual meetings and seminars. The continuing internationalization of the field will be essential as neuroscience research and its use become increasingly multi-national, multi-cultural and multi-focal in scope and effect.

To accommodate this momentum, programs in neuroethics have been created within several universities, think tanks, and governmental agencies; these include:

I. Asia

a. ELSI branch of Taiwan National Research Program for Genomic Medicine (NPPGM)

b. Neuroethics Working Group of the Bioethics Advisory Committee at the National University of Singapore (https://www.bioethics-singapore.org/index.php/activities/current-projects)

c. Research Institute of Science and Technology for Society (RISTEX) (subsection of Japan Science and Technology Agency 2004 (http://www.ristex.jp/EN/aboutus/history.html) http://www.ristex.jp/EN/past/brain/index.html (R&D Focus Area 2001-2009)

d. Strategic Research Program of Brain Sciences (SRPBS), Ministry of Education, Culture, Sports, Science, and Technology, RIKEN Brain Science Institute, Japan (https://nijc.brain.riken.jp/modules/projects/index.php?content_id=10&ml_lang=en)

II. Australia/New Zealand

a. Moral Cognition, Neuroethics, and Neurolaw Research Cluster at CAVE (The Centre for Agency, Values, and Ethics) at Macquarie University, (http://cave.mq.edu.au/home/)

b. The University of Queensland Centre for Clinical Research (UQCCR) Neuroethics, University of Queensland, Brisbane, St. Lucie Queensland (http://www.uqccr.uq.edu.au/neuroethics)

III. North America

a. Center for Cognition and Neuroethics, Flint, MI, USA (*Joint affiliation between the Department of Philosophy at the University of Michigan-Flint and the Insight Institute of Neurosurgery and Neuroscience (IINN) (http://cognethic.org/)*

b. Center for Neuroscience and Society of University of Pennsylvania, Philadelphia, PA, USA (http://www.neuroethics.upenn.edu/)

c. Center for Neurotechnology Studies at the Potomac Institute for Policy Studies, Arlington, VA, USA (http://www.potomacinstitute.org/)

d. Cognitive Neuroscience Society, Center for Mind and Brain, Davis, California, USA (http://www.cogneurosociety.org/about/)

e. Mind, Brain Imaging, and Neuroethics Unit, University of Ottawa, Ottawa, Canada (http://www.imhr.ca/research/mind-neuroethics-e.cfm)

f. Montreal Neuroethics Network, McGill University, Montreal, Canada (http://www.mcgill.ca/psychiatry/category/tags/montreal-neuroethics-network)

g. National Core for Neuroethics, University of British Columbia, Vancouver, Canada (http://neuroethics.med.ubc.ca/)

h. Neuroethics Research Unit, Institut De Recherche Clinique De Montreal, Montreal, Canada (http://www.ircm.qc.ca/LARECHERCHE/axes/neuro/neuroethique/Pages/index.aspx?PFLG=1033)

i. Neuroethics Studies Program, Pellegrino Center for Clinical Bioethics, Georgetown University Medical Center, Washington DC, USA (https://clinicalbioethics.georgetown.edu/neuroethicsprogram)

j. Neuroethics New Emerging Team (NET) Dalhousie University, Dalhousie, Nova Scotia (http://www.neuroethics.ca)

k. NeuroEthics Program at Cleveland Clinic, Cleveland, OH, USA (http://my.clevelandclinic.org/about-cleveland-clinic/ethics-humanities-care/neuroethics.aspx)

l. Program in Ethics and Brain Sciences (PEBS), Baltimore, MD, USA (*Joint affiliation between at Johns Hopkins Berman Institute of Bioethics Neuroethics Program and Johns Hopkins Brain Sciences Institute) (http://www.bioethicsinstitute.org/research/science-ethics/program-in-ethics-and-brain-sciences )*

m. Program in Neuroethics Stanford Center for Biomedical Ethics, Stanford, CA, USA. (http://neuroethics.stanford.edu/)

IV. South/Central America

a. Grupo de Pesquisa em Neurofilosofia – PUCRS, Instituto do Cérebro, Brazil

b. Programa de Estudios en Neuroética: Centro de Investigaciones Filosóficas (CIF), Argentina (http://programaneuroeticacif.wordpress.com/)

c. Neuroética, filosofia experimental e filosofia da mente, Brazil (http://plsql1.cnpq.br/buscaoperacional/detalhegrupo.jsp?grupo=0009701KU1BMW3)

d. NeuroEduc—Núcleo de Estudos em Neurociências e Educação—Grupo de Pesquisa e Desenvolvimento—CNPQ, Brazil (http://www.cienciasecognicao.org/portal/?page_id=66)

e. Mente, Ética e Póshumanismo (MEPH), Universidade Federal do Piaul—UFPI, Brazil (http://plsql1.cnpq.br/buscaoperacional/detalhegrupo.jsp?grupo=0323701LX6ENZ9)

V. United Kingdom

a. Centre for Cognitive Liberty and Ethics, Cardiff University, Cardiff, UK. (http://www.cognitiveliberty.org/proj_neuro.html)

b. Oxford-Wellcome Centre for Neuroethics, University of Oxford, Oxford, UK (http://www.neuroethics.ox.ac.uk/)

VI. Western Europe

a. Ethik in der Praxis, Ruhr-Universität Bochum, Bochum, GER (http://www.ruhr-uni-bochum.de/malakow/klin_ethik/ethikkomitees_gruendung.html)

b. Institut de Cerveau et de la Moelle Epinière (ICM), Paris, FR (http://icm-institute.org/menu/actualites)

c. Munich Center for Neurosciences, the Ludwig-Maximilians University of Munich, GER (http://www.gsn.uni-muenchen.de/download/general/mcn_brosch_fuer_internet.pdf)

d. Italian Society for Neuroethics (Società Italiana di Neuroetica), San Raffaele University, in Milan, IT (http://societadineuroetica.it)

e. Research Group on Neuroethics and Neurophilosophy, Johannes Gutenberg University of Mainz, Mainz, GER (https://teamweb.unimainz.de/fb05/Neuroethics/SitePages/Home.aspx)

f. Science, Ethics, and Society Initiative, Munich, GER (Joint affiliation of Technical University of Munich and the Ludwig-Maximilians University of Munich)

g. Neuroethics Research Group, World Federation of Neurology (http://wfneurology.org/researchGroups.php)

Many inter-disciplinary programs integrating biology, psychology, and cognitive science allow for students to study, and focus advanced scholarship, in neuroethics. In addition, numerous universities offer courses or workshops in neuroethics, as the medical, legal, and social issues of neuroethics tend to attract students from a variety of fields. Other than a few exceptions, such as the University of British Columbia, University of Pennsylvania, and Georgetown University, there are no programs of study specifically dedicated to neuroethics. In response, the program at the University of Pennsylvania provides links to Open Educational Resources (https://sites.sas.upenn.edu/neuroethics) that afford course materials (such as syllabi and useful links on the website) that encourage and support teachers and professors (of all levels and disciplines) to embrace neuroethics.

## Aims

**Figure 1:**
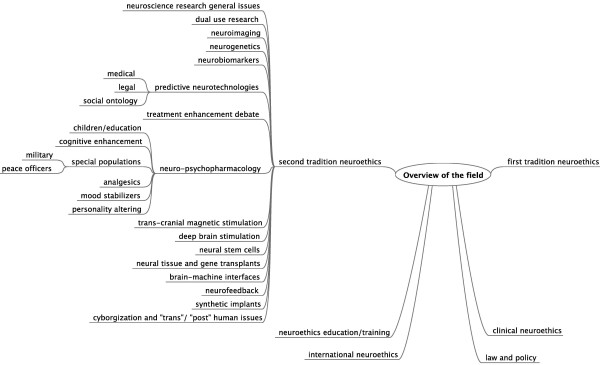
Diagrammatic representation of domains of neuroethics as a discipline.

Given the growth, inter-disciplinarity, and rapid maturation of the field of neuroethics since 2002 (see Figure 1), we posit the need for, and utility of a comprehensive bibliography of the neuroethics’ literature. Toward this end, we provide an iterative, four-part document that affords a repository of international papers, books, and chapters that address the field in overview, and present discussion(s) of more particular aspects and topics of neuroethics. As shown in Table 1, this first installment lists reviews and overviews of the discipline, and broad summaries of basic developments and issues of the field. Also included are reflections upon the history and multicultural perspectives of neuroethics. Part I will be followed by three installments, to address specific topics in neuroethics.

**Table 1:** Scope and contents of four-part neuroethics bibliography

	
**PART I**	**Overviews**
	**Reviews**
	**Historical descriptions of neuroethics**
	**Definitions and depictions of the field and its practices**
**PART II**	**“First tradition neuroethics:” The neuroscience of morality and ethics**
	**Neuroscientific studies of moral cognition, emotion, decision-making, and behavior**
	**Neuroscientific studies of “free will”**
	**Neuroscientific studies of egoism and altruism**
	**Implications of first tradition neuroethics**
**PART III**	**“Second tradition neuroethics:” Specific ethical issues in neuroscience**
	**Research issues**
	**Translational models and problems**
	**Use of animal models in neuroscience/animal neuroethics**
	**Clinical neuroethics**
	**Treatment, enablement, enhancement**
	**Use of novel neuroscientific and neurotechnological approaches in medicine**
	**● Neurology**
	**● Psychiatry/Psychology**
	**● Pain care**
	**● Rehabilitative medicine/prosthetics**
**PART IV**	**Neuroethics and society/ international neuroethics**
	**Multicultural issues and perspectives in neuroethics**
	**Studies of multinational approaches in/to neuroethics**
	**Neuroethics, law, public safety, and national security and defense**
	**Neuroethics and policy**
	**Neuroethics education/training**

## Methods

Literature devoted to neuroethics can be found in books, journals, and web documents addressing medicine, biosciences, engineering, philosophy and other humanities, law, and the social sciences, as well as in general reference works and databases such as WorldCat (see below). To systematically survey the neuroethics literature, searches were performed in the following databases:

1. PubMed (http://pubmed.gov): This is the U.S. National Library of Medicine’s (NLM) publicly accessible bibliographic database of journal articles from medicine, the life sciences and selected titles from the humanities;

2. The NLM Catalog (http://www.ncbi.nlm.nih.gov/nlmcatalog); a publicly accessible bibliographic database of books, book chapters, digital documents, and videos. The databases cover items in 60 languages that are indexed using MeSH (Medical Subject Headings). A subset of PubMed records is linked to full-text articles in the open access digital repository PubMed Central (http://www.ncbi.nlm.nih.gov/pmc/). In cases where funding requirements mandate open access publishing, selected articles from journals are made available through PubMed Central. For example, the only articles from *AJOB Neuroscience* available through PubMed are PubMed Central documents.

3. Academic Search Premier. Presenting full-text compendia of academic journals from a wide range of disciplines including the basic sciences, social sciences, and the humanities, it covers documents in 22 languages from 1965 to the present (NB: some earlier coverage may also be provided). This proprietary database is produced by EBSCO Information Services*. AJOB Neuroscience* is fully indexed in Academic Search Premier.

4. Proquest Research Library. Affording a full-text database of books, conference proceedings, government documents, magazines, newspapers, pamphlets, scholarly journal articles, audio and video works and wire feeds, this resources provides documents in 42 languages from 1971 to the present. An example of a rare neuroethics document identified via this database is a transcript of a National Public Radio (NPR) segment featuring Henry Greely discussing the use of neuroimaging in the law. This proprietary database is produced by Proquest LLC, a subsidiary of Cambridge Information Group.

5. JSTOR. Comprised of three components: (a) a full-text archive of scholarly journals, (b) a digital library of current journal titles, and (c) digitized primary resources including books and reports, this resource affords US literature published prior to 1923, inclusive of titles such as the *Philosophical Transactions of the Royal Society* (UK) issues of which are available since its inception in 1665. Neuroethics literature found in JSTOR includes news items and editorials that are not indexed in other databases such as PubMed. JSTOR is a proprietary database produced by ITHAKA, an academic library services provider.

6. LexisNexis Academic. While primarily a full-text legal database, LexisNexis also provides access to a wide variety of documents such as U.S. Congressional hearings, news items, wire service reports, television and radio transcripts, international magazines, blog posts and web publications. United States’ Supreme Court decisions and patents are available from 1790 to the present. International case law is included for the European Union and a number of other countries. A wide range of newspaper articles on neuroethics topics can be retrieved, including posts from *The Guardian* (U.K.) and *India Pharma News* (New Delhi.) LexisNexis is a proprietary database produced by Reed Elsevier.

7. WorldCat (http://www.worldcat.org). A combined library catalog of more than 2 billion records from over 72,000 libraries worldwide, WorldCat is publicly accessible, and contains records for books, audiovisual materials, journal articles, musical scores, and web documents. Records for neuroethics items can be retrieved in over 40 languages.

8. Philosopher’s Index. As a bibliographic database covering the philosophical literature, predominately journal articles but also some books, the database includes citations and abstracts in English, French, German, Italian and Spanish, and covers documents written in 39 languages from 1940 to the present. Examples of international journals featuring articles on neuroethical issues are the Lithuanian journal *Mokslo darbai: Problemos* (Problems: Research Papers), and *Isegoría: Revista de Filosofía Moral y Política* published by the Consejo Superior de Investigaciones Cientificas (CSIC) (Spanish National Research Council.) Philosopher’s Index is a proprietary database produced by the Philosopher’s Information Center.

9. Embase. A bibliographic database focusing on the biomedical literature that includes journals from over 70 countries. Coverage dates from 1947 to the present. Embase is a proprietary database produced by Elsevier. Articles from the journal *Neuroethics* are indexed in Embase.

10. BELIT (http://www.drze.de/belit/recherche/schnellsuche/recherche.html). Providing a publicly accessible database, BELIT brings together online resources from five ethics centers: IDEM (Information and Documentation Centre on Ethics in Medicine) at the Academy for Ethics in Medicine in Göttingen, GER; IZEW (International Centre for Ethics in the Sciences and Humanities) at the University of Tübingen, GER; CDE (Centre de Documentation en Éthique) at the Comité Consultatif National d'Éthique, Paris, FRA; KIE (Kennedy Institute of Ethics) at Georgetown University, Washington, DC, USA; and DRZE (German Reference Centre for Ethics in the Life Sciences) in Bonn, GER. DRZE coordinates the project. Entering terms in English, German or French will retrieve citations in all three languages due to the utilization of a multilingual bioethics thesaurus.

11. Web of Knowledge/Web of Science (WoS). A proprietary database produced by Thomson Reuters, WoS combines over 20 databases with some back-files dating from 1900. The “core databases” are: Arts and Humanities Citation Index, Conference Proceedings Citation Index, Science Citation Index Expanded, and Social Sciences Citation Index Expanded. In addition, WoS contains regional databases such as Brazil’s SciELO Citation Index and the Chinese Science Citation Database. Searches for neuroethical topics can be limited to conference proceedings, book chapters, or meeting abstracts.

Open access repositories were another source of documents addressing topics in neuroethics that were utilized. These repositories included:

1) Digital Public Library of America (DPLA) (http://dp.la/) A collaborative effort of U.S. academic and public libraries led by the Berkman Center for Internet & Society at Harvard University. DPLA provides full-text access to public domain documents, electronic books, and online journals such as *Trends in Neurosciences*.

2) Directory of Open Access Journals (DOAJ) (http://www.doaj.org/) A collaborative project of both non-profit open access publishers such as BioMed Central and the open access divisions of for-profit publishers such as Taylor and Francis. Articles on neuroethics can be retrieved by text-word searches of titles and keywords.

3) Hathi Trust Digital Library (http://www.hathitrust.org/) A project of US university libraries coordinated by the University of California. The digital library contains public domain documents pertinent to neuroethical issues such as U.S. House of Representatives’ committee hearings.

4) European Library (http://www.theeuropeanlibrary.org/tel4/) A cooperative catalog of over 45 European libraries with some full-text access to books, conference proceedings and journals addressing neuroethical issues.

5) Internet Archive (http://archive.org/). A digital library maintained by a non-profit organization headquartered in San Francisco, California (USA). The archive contains audio and video files pertaining to neuroethics.

6) Globethics.net (http://www.globethics.net/) Produced by a global network of ethicists and ethics institutions, Globalethics.net was organized at the 2003 World Summit on the Information Society (WSIS), a UNESCO meeting held in Paris each year. It includes links to journals such as *The Internet Journal of Law, Healthcare and Ethics* that contain articles on neuroethical issues.

7) Neuroethics-Wikiography (https://teamweb.uni-mainz.de/fb05/Neuroethics/) An online bibliography of journal articles on neuroethics from 2004 to the present. The site is produced by the Research Group on Neuroethics/Neurophilosophy, Department of Philosophy, Johannes Gutenberg-Universität Mainz, GER.

8) Law and Neuroscience Bibliography (http://www.lawneuro.org/bibliography.php) is maintained by the Research Network on Law and Neuroscience at Vanderbilt University, TN, USA.

Several open access bioethics journals were not contained in the Directory of Open Access Journals (DOAJ) and therefore were searched individually; these were:

1) *Journal of Ethics and Social Philosophy* from the University of Southern California (http://www.jesp.org/);

2) *Journal of Mental Health Ethics* from McMaster University (http://www.jemh.ca/). The supplement for volume 6 (2011/2012) is devoted to neuroethics;

3) *Journal of Practical Ethics* (http://www.jpe.ox.ac.uk/) from the Oxford Uehiro Centre for Practical Ethics at the University of Oxford; and

4) *Philosophers’ Imprint* from the University of Michigan (http://www.philosophersimprint.org/).

Citations obtained from open access journals were reviewed to determine if they have been published in journals abiding by the Committee on Publication Ethics (COPE) guidelines, or were indexed in one of the databases listed above. Only those that were published in COPE-referent journals and database indexed were included in the bibliography.

## Search strategies

The indexing language for NLM’s databases, MeSH (Medical Subject Headings) (http://www.ncbi.nlm.nih.gov/mesh/) provided the basic search strategy for each topic. In addition to clinical terminology, MeSH contains ethics-related terms developed for BIOETHICSLINE, a specialty database devoted to bioethical issues produced for NLM by the Kennedy Institute of Ethics from 1975–2000. BIOETHICSLINE records were incorporated into PubMed and NLM Catalog in 2000.

## Overlapping coverage

A number of core bioethics journals have been indexed in PubMed from their inception. These include: *Accountability in Research*; *AJOB* (American Journal of Bioethics); *American Journal of Law and Medicine*; *Bioethics*; *Cambridge Quarterly of Healthcare Ethics*; *European Journal of Health Law*; *Journal of Bioethical Inquiry*; *Journal of Clinical Ethics*; *Journal of Health Politics, Policy and Law*; *Journal of Legal Medicine*; *Journal of Medical Ethics*; *Journal of Medicine and Philosophy*; *Kennedy Institute of Ethics Journal*; *Nursing Ethics*, and *Science and Engineering Ethics*.

In addition, PubMed includes significant coverage of journals such as *Developing World Bioethics*, *Monash Bioethics Review*, and *Theoretical Medicine and Bioethics*. The percentage of overlap with other databases, such as Philosopher’s Index and Academic Search Premier, ranges between 50% - 60%. In developing the bibliography, the *RefWorks* citation manager program was utilized to eliminate duplicate reference citations.

The following references are to articles, book chapters, and monographs that provide overviews/reviews of neuroethics as a field of study.

Review Articles

• _____. **Neuroethics: an overview**. *Biotechnol J* 2008, **3**(12):1467–1468. doi:10.1002/biot.200890110.

• Abi-Rached JM: **The implications of the new brain sciences**. *EMBO Rep* 2008, **9**(12):1158–1162. doi:10.1038/embor.2008.211.

• Arnaudo L: **Diritto cognitivo. Prolegomeni a una ricerca**. *Politica del Diritto* 2010, **41**(1):101–136.

• Ashcroft R: **Ethics of neuroscience or neuroscience of ethics**? *Lancet Neurol* 2006, **5**(3):211. doi:10.1016/s1474-4422(06)70371-4.

• Ashcroft R: **The social side of neuroethics**. *Lancet Neurol* 2005, **4**(2):85. doi:10.1017/S146114571100037X.

• Baertschi B: **Neurosciences and neuroethics**. *Rev Med Suisse* 2005, **1**(34):2225–2229.

• Benanti P: **From Neuroskepticism to neuroethics: role of morality in neuroscience that becomes neurotechnology**. *AJOB Neurosci* 2010, **1**(2):39–40. doi:10.1080/21507741003699264.

• Boella L: **Out of our minds: il profilo innovativo della neuroetica[the innovative profile of neuroethics]**. *Giornale Italiano di Psicologia* 2010, **4**:787–792. doi:10.1421/33419.

• Braude HD: **Evaluating moral intuitions in neuroethics: a neurophenomenological perspective**. *AJOB Neurosci* 2011, **2**(2):22–24.

• Brosnan C: **The sociology of neuroethics: expectational discourses and the rise of a new discipline**. *Sociology Compass* 2011, **5**(4):287–297. doi:10.1111/j.1751-9020.2011.00365.x.

• Bruni T, Boniolo G: **Neuroetica e balcanizzazione della bioetica**. *Giornale italiano di psicologia,* 2010, **37**(4):793–798.

• Buller T: **What can neuroscience contribute to ethics?***J Med Ethics* 2006, **32**(2):63–64. doi:10.1136/jme.2005.014506.

• Cabrera L: **Neuroethics: a new way to do ethics or a new understanding of ethics**? *AJOB Neurosci* 2011, **2**(2):25–26.

• Caffo L: **Neuroetica in Italia: una rassegna filosofica**. *Rivista Internazionale di Filosofia e Psicologia* 2013, **4**(3):385–389.

• Chahal HS, Chen DT: **Neuroethics: challenge for the 21st century**. *J Neuroophthalmol* 2009, **29**(2):166–167. doi:10.1097/01.wno.0000354348.14270.29.

• Cheshire WP, Jr: **Neuroscience, nuance, and neuroethics**. *Ethics & Medicine: An International Journal of Bioethics* 2006, **22**(2):71–3.

• Cheung, EH: **A new ethics of psychiatry: neuroethics, neuroscience, and technology**. *J Psychiatr Pract* 2009, **15**(5):391–401. doi:10.1097/01.pra.0000361279.11210.14.

• Chneiweiss H: **Spécificité des dilemmes éthiques résultant des travaux développés en neurosciences: émergence d’une neuroéthique**. *PSN* 2005, **3**(13):150–157. doi:10.1007/BF03005835.

• Clausen J: **Neurotechnologien interdisziplinär: anthropologische und ethische Überlegungen**. LIFIS Online 2011:1–9. http://www.leibniz-institut.de/archiv/clausen_20_03_11.pdf.

• Clausen J, Mueller O, Schwenzfeur, S: **Neuroethik-aktuelle faragen im spannungsfeld zwischen neurowissenschaft und ethic.***Zeitschrift fur Evangelische Ethik* 2008, **52**(4):286–297.

• Comerford K, Rasmussen E, Illes J: **Evidence of a new and evolving discipline: neuroethics literature, 2002–2007**. In *Proceedings of the American Society for Information Science and Technology.* Vancouver, Canada; 2009, **46**(1):1–5. doi:10.1002/meet.2009.14504603113.

• Conrad E, De Vries, R: **Field of dreams: a social history of neuroethics**. *Adv Med Sociol* 2003, **13**:299–324.

• Cortina A: **Neuroética: Las bases cerebrales de una ética universal con relevancia política [Neuroethics: the brain’s foundations for politically relevant ethics?]**. *Isegoría* 2010, **42**:129–148.

• Cubelli R, De Bastiani P: **La (neuro) etica del perito**. *Giornale Italiano di Psicologia* 2010*,***37**(4):805–810.

• De Vries R: **Who will guard the guardians of neuroscience? Firing the neuroethical imagination**. *EMBO Rep* 2007, **8(**1):S65-S69. doi:10.1038/sj.embor.7401010.

• De Vries R: **Framing neuroethics: A sociological assessment of the neuroethical imagination**. *Am J Bioeth* 2005, **5**(2):25–27. doi:10.1080/15265160590960267

• Dranseika V, Noreika S, Gefenas E: **Neuroetikos žemėlapos**. *Problemos* 2009, **76**: 66–73.

• Evers K: **Philosophical challenges in neuroethics**. *Eur Neuropsychopharmacol* 2008, **18**:S202. doi:10.1016/s0924-977x(08)70234-7.

• Evers K: **Towards a philosophy for neuroethics: An informed materialist view of the brain might help to develop theoretical frameworks for applied neuroethics**. *EMBO Rep* 2007, **8**(1S):S48-S51. doi:10.1038/sj.embor.7401014.

• Farah MJ: **Emerging ethical issues in neuroscience**. *Nature Neuroscience* 2002, **5**(11):1123–1129. doi:10.1038/nn1102-1123.

• Farah MJ: **Neuroethics: The practical and the philosophical**. *Trends in Cogn Sci* 2005, **9**(1):34–40. doi:10.1016/j.tics.2004.12.001.

• Farah MJ: Neuroethics: a guide for the perplexed. *Cerebrum* 2004, 6(4):29–38.

• Farah MJ: **Neuroethics: the ethical, legal, and societal impact of neuroscience**. *Annu Rev Clin Psychol* 2012, **63**:571–591. doi:10.1146/annurev.psych.093008.100438.

• Farah MJ, Wolpe PR. **Monitoring and manipulating brain function: new neuroscience technologies and their ethical implications.***Hastings Cent Rep* 2004, **34**(3):35–45.

• Fergusson A: **Neuroethics: the new frontier**. *Ethics & Medicine: An International Journal of Bioethics* 2007, **23**(1):31–33.

• Figueroa G: **Neuroética: reflexiones sobre los principios latentes de la moral en medicine**. *Revista Médica de Chile* 2012, **140**(8):1078–1084.

• Fins JJ: **A leg to stand on: Sir William Osler and Wilder Penfield’s “Neuroethics.”***AJOB* 2008, **8**(1):37–46. doi:10.1080/15265160701841975.

• Fins JJ, Shapiro ZE: **Neuroimaging and neuroethics: clinical and policy considerations**. *Curr Opin Neurol* 2007, **20**(6):650–654. doi:10.1097/wco.0b013e3282f11f6d

• Focquaert F: **Bioethics and the Brain**. *Philos Psychol* 2009, **22**(3):397–401.

• Fuchs T: **Ethical issues in neuroscience**. *Curr Opin Psychiatry* 2006, **19**(6):600–607. doi:10.1097/01.yco.0000245752.75879.26.

• Fukushi T, Sakura O: **Exploring the origin of neuroethics: from the viewpoints of expression and concepts**. *AJOB* 2008, **8**(1):56–57. doi:10.1080/15265160701839672

• Garcia-Morales I: **Neuroethics**. *Neurologia* 2009, **5**(1):12–15.

• Gassen HG. **Why neuroethics?***Biotechnol J* 2008, **3**(12):1463–1465. doi:10.1002/biot.200890109.

• Gillett G: **Neuroethics, Neo-Lockeanism, and Embodied Subjectivity**. *Philos Psychiatr Psychol* 2013, **20**(1): 43–46.

• Gillett G: **The subjective brain, identity, and neuroethics**. *AJOB* 2009, **9**(9):5–13.

• Giordano J: **Neuroethics: traditions, tasks and values**. *The Human Prospect* 2011, **1**(1):2–8.

• Giordano J: **Neuroethics: interacting “traditions” as a viable meta-ethics**. *AJOB Neurosci* 2011, **2**(2):17–19. doi:10.1080/21507740.2011.559922.

• Giordano J: **Unpacking neuroscience and neurotechnology – instructions not included: neuroethics required**. *Neuroethics* 2013, **6**(2):411–414. doi:10.1007/s12152-011-9150-4.

• Giordano J: **The human prospect(s) of neuroscience and neurotechnology: Domains of influence and the necessity – and questions – of neuroethics**. *The Human Prospect* 2014, 3(3): 2–18.

• Giordano J, Benedikter R: **An early—and necessary—flight of the Owl of Minerva: neuroscience, neurotechnology, human socio-cultural boundaries, and the importance of neuroethics.***J Evol Technol* 2011, **22**(1):110–115.

• Giordano J, Olds J: **On the interfluence of neuroscience, neuroethics and legal and social issues: the need for (N)ELSI**. *AJOB Neurosci* 2010, **1**(4):12–14. doi:10.1080/21507740.2010.515964.

• Glannon W: **Neuroethics**. *Bioethics* 2006, **20**(1):37–52. doi:10.1111/j.1467-8519.2006.00474.x.

• Glannon W: **Our brains are not us**. *Bioethics* 2009, **23**(6):321–329. doi:10.1111/j.1467-8519.2009.01727.x.

• Greely HT: **Neuroethics: where do we go from here**? *AJOB Neurosci* 2010, **1**(3):1–2. doi:10.1080/21507740.2010.490156.

• Greely HT: **What if? the farther shores of neuroethics**. *Sci Eng Ethics* 2012, **18**(3):439–446. doi:10.1007/s11948-012-9391-6.

• Greely HT: **Neuroethics and ELSI: similarities and differences**. *Minn J Law Sci Technol* 2006, **7**(2):599–777.

• Häyry M: **Neuroethical theories**. *Cambrid Q Healthc Ethics* 2010, **19**(2):165–78. doi:10.1017/S0963180109990430.

• Hu J, Mao C: **Neuroethics: the perfect intersections of humanities and neuroscience**. *Jiangxi lan tian xue yuan xue bao/Journal of Jiangxi Blue Sky University* 2008, 2.

• Hu J, Wang T, Yin J, Wang Y. **Neuro-ethics: a research report from Chinese investigation**. In *Proceedings of SPIE: 2011 International Conference on Photonics, 3D-Imaging, and Visualization.* Edited by Zhu E. SPIE: Bellingham, Washington; 2011, **82053**:156–160. doi:10.1117/12.906102.

• Huber L: **Jens Clausen (2011). Technik im gehirn: ethische, theoretische und historische Aspekte moderner neurotechnologien**. *Ethik Med* 2013, **25**(4):373–374. doi:10.1007/s00481-013-0255-7.

• Illes J: **Brain science and public discourse.***Cerebrum*, **4**(3):68–70.

• Illes J: **Empowering brain science with neuroethics**. *Lancet Neurol* 2010*,***376**(9749):1294–1295. doi:10.1016/s0140-6736(10)61904-6.

• Illes J, Bird SJ: **Neuroethics: A modern context for ethics in neuroscience**. *Trends Neurosci* 2006, **29**(9):511–517. doi:10.1016/j.tins.2006.07.002.

• Illes J, Raffin TA: **Neuroethics: an emerging new discipline in the study of brain and cognition**. *Brain and Cognition* 2002, **50**(3):341–344.

• Illes J, Blakemore C, Hansson MG, et al.: **International perspectives on engaging the public in neuroethics**. *Nat Rev. Neurosci* 2006, **6**(12):977–82. doi:10.1038/nrn1808.

• Illes J, Bird S: **Neuroethics: a modern context for ethics in neuroscience**. *Trends Neurosci* 2006, **29**(9):511–517. doi:10.1016/j.tins.2006.07.002.

• Illes J: **Empirical neuroethics. Can brain imaging visualize human thought? Why is neuroethics interested in such a possibility?***EMBO Rep* 2007, **8**(1):S57-S60.

• Illes J, et al.: **International perspectives on engaging the public in neuroethics**. *Nat Rev. Neurosci* 2005, **6**(12): 977–982. doi:10.1038/nrn1808.

• Illes J, Racine E: **Neuroethics: dialogue on a continuum from tradition to innovation**. *AJOB* 2005, **5**(2): W3-W4. doi:10.1080/15265160590969114.

• Jácomo A: **Novos desafios bioéticos em neurociência** [New bioethical challenges in neuroscience]. *Revista Bioethikos* 2013, **7**(1):27–35.

• Jančić E, Katić-Bubaš J, Bradić N: **Neuroetika**, *Medicina* 2008, **44**(2):186–190.

• Justo L, Erazun F: **Neuroethics needs an international human rights deliberative frame**. *AJOB Neurosci* 2010, **1**(4):17–18. doi:10.1080/21507740.2010.515559.

• Kalichman M, Plemmons D, Bird S: **Editors’ Overview: Neuroethics: many voices and many stories.***Sci Eng Ethics* 2012, **18**(3):423–432. doi:10.1007/s11948-012-9398-z

• Kagawa C: **Neuroethics and bioethics – implications of balkanization controversy**. *Brain and Nerve = Shinkei Kenkyu no Shinpo* 2009, **61**(1):11–17.

• Kipper DJ: **Neuroethics: a methodological reflection**. *Revista Bioetica* 2011, **19**(1):29–43.

• Klein E: **Is there a need for clinical neuroskepticism?***Neuroethics* 2011, **4**(3):251–259. doi:10.1007/s12152-010-9089-x.

• Knoppers BM: **Neuroethics, new ethics.***AJOB* 2005, **5**(2):33, W3-33. doi:10.1080/15265160590960393.

• Lavazza A, Sartori G: **Neuroetica. Una nuova prospettiva di ricerca**. *Giornale Italiano di Psicologia* 2010, **37**(4):755–778.

• Lavazza A, De Caro M: **Neuroetica, la nascita di un nuovo tipo antropologia**? *Rivista Internazionale di Filosofia e Psicologia* 2013, **4**(3):252–263.

• Levy N: **Introducing neuroethics**. *Neuroethics* 2008, **1**(1):1–8. doi:10.1007/s12152-008-9007-7.

• Levy N: **Neuroethics: ethics and the sciences of the mind**. *Philos Compass* 2009, **4**(1):69–81. doi:10.1111/j.1747-9991.2008.00195.x.

• Levy N: **Neuroethics: a new way of doing ethics**. *AJOB Neuroscience* 2011 **2**(2):3–9. doi:10.1080/21507740.2011.557683.

• Levy N: **Rethinking neuroethics in the light of the extended mind thesis**. *AJOB* 2007, **7**(9):3–11. doi:10.1080/15265160701518466.

• Levy N, Clarke S: **Neuroethics and psychiatry**. *Curr Opin Psychiatry* 2008, **21**(6):568–571. doi:10.1097/YCO.0b013e3283126769.

• Lombera S, Illes J: **The international dimensions of neuroethics**. *Dev World Bioeth* 2009, **9**(2):57–64. doi:10.1111/j.1471-8847.2008.00235.x.

• Lyng S: **Brain, body, and society: bioethical reflections on socio-historical neuroscience and neuro-corporeal social science**. *AJOB* 2009, **9**(9):25–26. doi:10.1080/15265160903095982.

• Malafouris L: **More than a brain: human mindscapes**. *Brain* 2012, **135**(12):3839–3844. doi:10.1093/brain/aws063.

• Marcus SJ: **Neuroethics: Mapping the Field***.* In *Conference Proceedings: 13–14 May 2002; San Francisco*: Dana Press; 2002.

• Mauron A: **Neuroethics: what else is new?***Schweizer Archiv für Neurologie und Psychiatrie* 2010, **161**(8):286–289.

• Miller JP: **Whose brain, which ethics**? *Hypatia* 2010, **25**(3):618–624. doi:10.1111/j.1527-2001.2010.01120.x.

• Murphy ER, Illes J: **Neuroethics and psychiatry: new collaborations for emerging challenges**. *Psychiatric Annuals* 2007, **37**(12):798.

• Minerva F: **Neuroetica, a look at the development of the Italian debate on neuroethics**. *Neuroethics* 2013, **6**(1):1–4. doi:10.1007/s12152-012-9160-x.

• Moosa E: **Translating neuroethics: reflections from Muslim ethics**. *Sci Eng Ethics* 2012, **18**(3):519–528. doi:10.1007/s11948-012-9392-5.

• Morein-Zamir S, Sahakian BJ: **Neuroethics and public engagement training needed for neuroscientists.***Trends in Cogn Sci* 2010, **14**(2):49–51. doi:10.1016/j.tics.2009.10.007.

• Moreno JD: **Neuroethics: an agenda for neuroscience and society***. Nat Rev Neurosci* 2003, **4**(2):149–153. doi:10.1038/nrn1031.

• Nagel S, Neubauer N: **A framework to systematize positions in neuroethics**. *Essays in Philosophy* 2005, **6**(1):24.

• Nahra C: **Neuroscience of ethics: the state of art and the promises for the future**. *Ethic@* 2011, **10**(1):109–132. doi:10.5007/1677-2954.2011v10n1p109.

• Niebrój LT: **Neuroetyka: Nowa Jakosc Etyki Medycznej? [Neuroethics: New Quality of Medical Ethics?]***Roczniki Pomorskiej Akademii Medycznej W Szczecinie* 2013, **59**(1):130–136.

• Northoff G: **What is neuroethics? Empirical and theoretical neuroethics**. *Curr Opin Psychiatry* 2009, **22**(6):565–569. doi:10.1097/YCO.0b013e32832e088b.

• Northoff G, Witzel J, Bogerts B: **Neuroethics--a future discipline? [Was ist “Neuroethik”--eine Disziplin der Zukunft?]***Nervenarzt* 2006, **77**(1):5–11. doi:10.1007/s00115-005-1895-8.

• Nukala VN, Halal W: **Emerging neurotechnologies: Trends, relevance and prospects**. *Synesis: A Journal of Science, Technology, Ethics and Policy* 2010, **1**(1):G36 - G53.

• O’Connell G: **Tracking the impact of neuroethics**. *Cortex* 2011, **47**(1):1259–1260. doi:10.1016/j.cortex.2011.04.020.

• O’Connell BM: **Anticipating Ethics**. In *Electronics and the Environment: Proceedings of the IEEE International Symposium:* Scottsdale; 2006: 48–52. doi:10.1109/ISEE.2006.1650031.

• Parens E, Johnston J: **Does it make sense to speak of neuroethics? Three problems with keying ethics to hot new science and technology**. *EMBO Rep* 2007, **8**(1):S61-S64. doi:10.1038/sj.embor.7400992.

• Pigliucci M: **The new science of neuroethics**. *Skeptic* 2012, **17**(4):20–23, 64.

• Polard I: **High tech neuroscience, neuroethics and the precautionary principle**. *Asia-Pacific Perspectives on Ethics of Science and Technology* 2007:37–43.

• Polonioli A: **Recent trends in neuroethics: a selected bibliography**. *Etica & Politica* 2009, **11**(2):68–87.

• Racine E, Illes J: **Neuroethical responsibilities**. *Can J Neurol Sci* 2006, **33**(3):269–277.

• Racine E: **Interdisciplinary approaches for a pragmatic neuroethics**. *AJOB* 2008, 8(1):52–53. doi:10.1080/15265160701828444.

• Racine E: **Are the conditions in place for the development of a strong and interdisciplinary neuroethics**? *AJOB Neurosci* 2012, **3**(2):1–2. doi:10.1080/21507740.2012.666329.

• Racine E, Illes J: **Neuroethical responsibilities**. *Can J Neurol Sci* 2006, **33**(3):269–277.

• Raeymaekers P, Rondia K, Slob M: **Connecting Brains and Society: The Present and Future of Brain Science, What is Possible, What is Desirable.** In *Proceedings and Synthesis Report from the European Workshop: 23–23 April 2004*; Amsterdam, The Netherlands. Edited by Peter Raeymakers, Karin Rondia, Marjan Slob: King Baudouin Foundation; The Rathenau Institute; 2004.

• Ramey CH: **Neuroethics: challenges for the 21st century**. *Philos Psychol* 2010, **23**(1):125–129.

• Rasmusson A: **Neuroethics as a brain-based philosophy of life: the case of Michael S. Gazzaniga**. *Neuroethics* 2009, **2**(1):3–11. doi:10.1007/s12152-008-9024-6.

• Roeser S: **Intuitions, emotions and gut reactions in decisions about risks: towards a different interpretation of ‘neuroethics’**. *Journal of Risk Research* 2010, **13**(2): 175–190. doi:10.1080/13669870903126275.

• Rommelfanger K, Boshears P: **Ethical use of neuroscience**. *AJOB Neurosci* 2011, 2(2):19–21. doi:10.1080/21507740.2011.559919.

• Roskies A: **Neuroethics for the new millenium**. *Neuron* 2002, **35**(1):21–23. doi:10.1016/S0896-6273(02)00763-8.

• Sakura O: **A view from the Far East: neuroethics in Japan, Taiwan, and South Korea**. *East Asian Science, Technology and Society* 2012, **6**(3):297–301. doi:10.1215/18752160-1728026.

• Schmidt-Felzmann H: **“Neuroethik” -zur Beziehung zwischen Neurowissenschaft und Ethik**. In *Proceedings der GAP.5: Philosophie und/als Wissenschaft*: 22–26 September 2003; Bielefeld. Edited by Anna Braungart: mentis, Paderborn; 2005: 636–646.

• Slachevsky Ch A: **La neuroética:¿ Un neologismo infundado o una nueva disciplina?***Revista Chilena de Neuro-psiquiatría* 2007, **45**(1):12–15. doi:10.4067/s0717-92272007000100003.

• Shook JR, Giordano J: **A principled and cosmopolitan neuroethics: considerations for international relevance.***Philos Ethics Humanit Med* 2014, **9**(1):1–13. doi:10.1186/1747-5341-9-1.

• Schlindwein-Zanini R, Schlemper B: **Neuroethics and neuroscience**. *Contextos Clínicos* 2013, **6**(1):58–61.

• Synofzik M: **Interventionen zwischen Gehirn und Geist: eine ethische analyse der neuen m** ***ö*** **glichkeiten der Neurowissenschaften [Intervening between brain and mind: an ethical analysis of the new possibilities of the neurosciences]**. *Fortschr Neurol Psychiatr* 2005, **73(**10):596–604. doi:10.1055/s-2004-830292.

• Tagliagambe S: **L’etica e le neuroscienze [The ethics of neuroscience]**. *S &F Scienza e Filosofia* 2011, 5.

• Tieu M: **Neuroethics**. *Issues* 2009, **86**:20–23.

• Trucchio A: **Neuroetica: una visita guidata [Neuroethics: a guided tour].***S&F Scienza e Filosofia* 2011, 5.

• Tsomo KL: **Compassion, ethics, and neuroscience: neuroethics through Buddhist eyes**. *Sci Eng Ethics* 2012, **18**(3):529–537. doi:10.1007/s11948-012-9369-4.

• Weisberg DS, et al. **The seductive allure of neuroscience explanations**. *Cogn Neurosci* 2008, **20**(3):470–477. doi:10.1162/jocn.2008.20040.

• Wolpe PR: **Enhancing neuroethics**. *AJOB Neurosci* 2011, **2**(4):1–2. doi:10.1080/21507740.2011.620422.

• Wolpe PR: **The neuroscience revolution**. *Hastings Center Report* 2002, **32**(4):8.

• Wu KCC, Fukushi T: **Neuroethics in Taiwan: could there be a Confucian solution?***East Asian Science, Technology and Society: An International Journal* 2012, **6**(3):321–334. doi:10.1215/18752160-1727522.

• Zorzanelli R, Ortega F: **Cultura somática, neurociências e subjetividade contemporânea**. *Psicologia & Sociedade* 2011, **23**:30–36.

• **香川知晶**. [Neuroethics, a Historical Perspective.] *科学基礎論研究* 2008, **35**(2):87–92.

• 伊吹友秀**. “ニューロエシックスと生命倫理: いかなる意味でニューロエシックスはひとつの独立した学問領域でありうるか (<;研究報告> 倫理学者のためのニューロエシックス**).” [The Neuro Ethics] *実践哲学研究* 2007, **30**:95–116.

• 마키노, 에이지. “**문화연구와 ‘휴머니티’의 과제**.” [Cultural Studies and the ‘humanity’ of the project] *아시아문화연구* 2010, **18**:5–95.

Books

• Ackerman S: *Hard Science, Hard Choices: Facts, Ethics, and Policies Guiding Brain Science Today.* New York: Dana Press; 2006.

• Appiah A: *Experiments in Ethics.* Boston: Harvard University Press; 2008.

• Bernat JL: *Ethical Issues in Neurology.* Philadelphia: Lippincott Williams & Wilkins; 2008.

• Bickle J: *The Oxford Handbook of Philosophy and Neuroscience.* Oxford: Oxford University Press; 2009.

• Blank RH: *Brain Policy: How the New Neuroscience Will Change Our Lives and Our Politics.* Washington, DC: Georgetown University Press; 1999.

• Blank RH: *Intervention in the Brain: Politics, Policy, and Ethics*. Cambridge, MA: MIT Press; 2013.

• Boella L: *Neuroetica: La Morale Prima della Morale*. Milano: R. Cortina; 2008.

• Bonete PE: *Neuroética: Práctica Una Ética Desde El Cerebro.* Bilbao: Desclée de Brouwer; 2010.

• Cerroni A, Rufo F: *Neuroética: Tra Neuroscienze, Etica E Società.* Turin, Italia: UTET Università; 2009.

• Chatterjee A, Farah M: *Neuroethics in Practice*. Oxford: Oxford University Press; 2013.

• Chneiweiss H: *Neurosciences et Neuroéthique: Des Cerveaux Libres et Heureux.* Paris: Alvik; 2006.

• Craver CF: *Explaining the Brain: Mechanisms and the Mosaic Unity of Neuroscience*. New York: Oxford University Press; 2007.

• Evers K, Goldstein V: *Neuroética: Cuando La Materia Se Despierta.*. Buenos Aires: Katz; 2010.

• Farah MJ: *Neuroethics: An Introduction with Readings*. Cambridge, MA: MIT Press; 2010.

• Farisco M: *Uomo, Natura, Tecnica. Il Modello Postumanistico*. Zikkurat, Edizioni, Roma; 2009.

• Farisco M: *Filosofia delle Neurosience: Cervello, Mente, Persona*. Padova: Edizioni Messaggero; 2012.

• Fehrle J: *Herausforderung Biologie: Fragen an die Biologie-Fragen aus der Biologie*. Berlin: Münster Lit; 2010.

• Gava G: *Neuroscienze e Neuroetica: Storia e Sviluppi*. Padova: CLEUP; 2012.

• Gazzaniga MS: *The Ethical Brain: The Science of Our Moral Dilemmas*. New York: Harper Perennial; 2006.

• Gazzaniga MS: *The Cognitive Neurosciences*. Cambridge, MA: MIT Press; 2004.

• Gillett G: *Subjectivity and Being Somebody: Human Identity and Neuroethics*. Charlottesville: Imprint Academic; 2008.

• Giménez AJM, Sánchez-Migallón S: *De La Neurociencia a La Neuroética: Narrativa Científica Y Reflexión Filosófica.* Barañaín, Navarra: EUNSA; 2010.

• Giordano J, Gordijn B: *Scientific and Philosophical Perspectives in Neuroethics*. New York: Cambridge University Press; 2010.

• Giordano J: *Neurotechnology: Premises, Potential, and Problems*. Boca Raton: CRC Press; 2012.

• Glannon W: *Bioethics and the Brain*. Oxford; New York: Oxford University Press; 2007.

• Glannon W: *Brain, Body, and Mind: Neuroethics with a Human Face*. New York: Oxford University Press; 2011.

• Glannon W: *Defining Right and Wrong in Brain Science: Essential Readings in Neuroethics*. New York: Dana Press; 2007.

• Hildt E, Bölte S: *Neuroethik*. Series: UTB. München, Basel: E. Reinhardt; 2012.

• Hyman SE: *Les Progrès de la Recherche sur le Cerveau: La Neuroéthique Evolue, Mise à Jour 2007 [Advances in Brain Research: Neuroethics Evolution, 2007].* Lausanne: The European Dana Alliance for the Brain; 2007.

• Illes J: *Neuroethics: Defining the Issues in Theory, Practice, and Policy*. Oxford: Oxford University Press; 2006.

• Illes J, Sahakian BJ: *The Oxford Handbook of Neuroethics*. Oxford: Oxford University Press; 2013.

• Lavazza A, Sartori G: *Neuroetica*. Bologna: il Mulino; 2011.

• Littlefield MM, Johnson JM: *The Neuroscientific Turn: Transdisciplinarity in the Age of the Brain*. Ann Arbor: University of Michigan Press; 2012.

• Legrenzi P; Umilta C. *Neuromania: On the Limits of Brain Science*. Translated by Anderson F. Oxford: Oxford University Press; 2011.

• Levy N: *Neuroethics: Challenges for the 21st Century*. New York: Cambridge University Press; 2007.

• Nobuhara Y: *Nōshinkei Rinrigaku no Tenbo.* Tōkyō: Keisō Shobō; 2008.

• Racine E: *Pragmatic Neuroethics: Improving Treatment and Understanding of Mind-Brain*. Cambridge: MIT Press; 2010.

• Rees D, Rose S: *The New Brain Sciences: Perils and Prospects*. New York: Cambridge University Press; 2004.

• Rolls, ET: *Neuroculture: On the Implications of Brain Sci*ence. Oxford: Oxford University Press; 2012.

• Rose S: *The Future of the Brain: The Promise and Perils of Tomorrow’s Neuroscience*. Oxford: Oxford University Press; 2006.

• Satel SL, Lilienfeld SO: *Brainwashed: The Seductive Appeal of Mindless Neuroscience*. New York: Basic Books; 2013.

• Schleim S, Spranger TM,Walter H: *Von der.Neuroethik zum Neurorecht?* Göttingen: Vandenhoeck & Ruprecht; 2009.

• Sironi VA, Di Francesco, M: *Neuroetica: La Nuova Sfida Delle Neuroscienze.* Roma: Laterza; 2011.

• Tancredi L: *Hardwired Behavior: What Neuroscience Reveals about Morality*. New York: Cambridge University Press; 2005.

• Vogelsang F, Hoppe C, Geyer C: *Ohne Hirn Ist Alles Nichts: Impulse Für Eine Neuroethik.* Neukirchen-Vluyn: Neukirchener Verlag; 2008.

• 信原幸弘. 原塑. Nobuhara Y, Hara S: 脳神経倫理学の展望: 第1版 *[Neuroethics Outlook].* 1st edition. Tōkyō: Keisōshobō ed. 東京: 勁草書房; 2008.

Book Chapters

• Bernat JL: **Neuroethics**. In *Ethical Issues in Neurology*. Edited by Frances DeStefano, Leanne McMillan. Philadelphia: Lippincott Williams & Wilkins; 2008:495–510.

• Bird SJ: **Neuroethics**. In *Encyclopedia of Science, Technology, and Ethics*. Edited by Carl Mitcham. Detroit: Macmillan Reference USA; 2005:1310–1315.

• Chen D, Quirion R: **From the internationalization to the globalization of neuroethics: some perspectives and challenges**. In *The Oxford Handbook of Neuroethics*. Edited by Judy Illes, Barbara J. Sahakian. Oxford: Oxford University Press; 2013:823–834.

• Chneiweiss H: **Neurosciences et neuroéthique**. In *Traité de Bioéthique II: Soigner la Personne, Evolutions, Innovations Thérapeutiques*. Edited by Emmanuel Hirsch. Toulouse: Erès edition; 2010: 493–506.

• Colombetti E: **Etica delle neuroscienze [Ethics of neuroscience].** In *Il Controllo della Mente. Scienza ed Etica della Neuromodulazione Cerebrale*. Edited by Vittorio A. Sironi, Mauro Porta. Laterza: Roma-Bari; 2011:208–221.

• Farah MJ: **Neuroethics**. In *The Penn Center Guide to Bioethics*. Edited by Vardit Ravitsky, Autumn Fiester, Arthur L. Caplan. New York: Springer Publishing Co; 2009: 71–83.

• Farah MJ: **Emerging ethical issues in neuroscience**. In *Defining Right and Wrong in Brain Science: Essential Readings in Neuroethics*. Edited by Walter Glannon. New York: Dana Press; 2007:19–36.

• Farah MJ: **Neuroscience and neuroethics in the 21st century**. In *The Oxford Handbook of Neuroethics*. Edited by Judy Illes, Barbara J. Sahakian. Oxford: Oxford University Press; 2013: 761–782.

• Fischbach R, Mindes J: **Why neuroethicists are needed**. In *The Oxford Handbook of Neuroethics*. Edited by Judy Illes, Barbara J. Sahakian. Oxford: Oxford University Press; 2013:343–376.

• Gazzaniga MS: **Facts, fictions and the future of neuroethics**. In *Neuroethics: Defining the Issues in Theory, Practice, and Policy*, Edited by Judy Illes. Oxford: Oxford University Press; 2006:141–148.

• Glannon W: **Neuroethics**. In *Ethical Issues in Modern Medicine: Contemporary Readings in Bioethics*. Edited by Bonnie Steinbock, John D. Arras, Alex J. London. New York: McGraw-Hill Higher Education; 2009: 856–870.

• Giordano J: **Neuroethics: coming of age and facing the future**. In *Scientific and Philosophical Perspectives in Neuroethics*. Edited by James Giordano, Bert Gordijn. New York: Cambridge University Press; 2010: xxv-xxix.

• Giordano J, Benedikter R: **Neurotechnology, culture and the need for a cosmopolitan neuroethics**. In *Neurotechnology: Premises, Potential and Problems*. Edited by James Giordano. Boca Raton: CRC Press, 2012:231–239.

• Keebler JR et al. **Neuroethics: considerations for a future embedded with neurotechnology.** In *Neuroadaptive Systems: Theory and Applications.* Edited by Magdalena Fafrowicz. Boca Raton: Taylor& Francis; 2012:333.

• Kennedy D: **Neuroscience and neuroethics**. In *Defining Right and Wrong in Brain Science: Essential Readings in Neuroethics*. Edited by Walter Glannon. New York: Dana Press; 2007:58–60.

• Ladwig B: **Neuroethics**. *Encyclopedia of Neuroscience*. Edited by Marc D. Binder, Nobutaka Hirokawa, and Uwe Windhorst. Berlin: Springer; 2009:2648–2651.

• Lazzarini I: **Neuroethics: the new millennium view**. In *Educating for Moral Action: A Sourcebook in Health and Rehabilitation Ethics*. Edited by Ruth B. Purtilo, Gail M. Jensen, Charlotte B. Royeen. Philadelphia: F.A. Davis; 2005:147–157.

• Levy N: **Preface**. In *Scientific and Philosophical Perspectives in Neuroethics*. Edited by James Giordano, Bert Gordijn. New York: Cambridge University Press; 2010:xiii-xxii.

• Markič O: **Nevroetika**. In *Javna Etika in Integriteta.* Edited by Bećir Kečanović, Samo Uhan. Ljubljana, Slovenia: Komisija za Preprecevanje Korupcije, Plesko; 2012:105–116.

• Reichlin M: **Neuroethics**. In *A Companion to Moral Anthropology*. Edited by Didier Fassin. Malden: Wiley-Blackwell; 2012:595–610.

• Moreno JD: **Neuroethics: an agenda for neuroscience and society**. In *Is There an Ethicist in the House? On the Cutting Edge of Bioethics*. Edited by Jonathan D. Moreno. Bloomington: Indiana University Press; 2005:219–233.

• Racine E, Illes J: **Neuroethics**. In *The Cambridge Textbook of Bioethics*. Edited by Peter A. Singer, A.M. Viens. New York: Cambridge University Press; 2008:495–504.

• Racine E, Zimmerman E: **Pragmatic neuroethics and neuroscience’s potential to radically change ethics**. In *The Neuroscientific Turn: Transdisciplinarity in the Age of the Brain*. Edited by Melissa M. Littlefield, Jenell M. Johnson. Ann Arbor: University of Michigan Press; 2012:135.

• Racine E, Forlini C: **Neuroethics: an emerging field of scholarship and practice**. In *Toward a Moral Horizon: Nursing Ethics for Leadership and Practice*. 2^nd^ edition. Edited by Janet L. Storch, Patricia Rodney, Rosalie Starzomski. Toronto: Pearson Press; 2013:509–526.

• Rolls E: **Neuroethics**. In *Neuroculture: On the Implications of Brain Science*. Edited by Edmund Rose. Oxford: Oxford University Press; 2012:297–307.

• Rose S: **Ethics in a neurocentric world**. In *The Future of the Brain: The Promise and Perils of Tomorrow's Neuroscience*. Edited by Steve Rose. Oxford: Oxford University Press; 2006:297–306.

• Rose S: **Introduction: the new brain sciences**. In *The New Brain Sciences: Perils and Prospects*. Edited by Dai Rees, Steven Rose. New York: Cambridge University Press; 2004:3–14.

• Roskies A: **Neuroethics for the new millennium**. In *Defining Right and Wrong in Brain Science: Essential Readings in Neuroethics*. Edited by Walter Glannon. New York: Dana Press; 2007:12–18.

• Safire W: **Visions for a new field of “neuroethics.”** In *Defining Right and Wrong in Brain Science: Essential Readings in Neuroethics*. Edited by Walter Glannon. New York: Dana Press; 2007:7–11.

• Whitehouse P: **A clinical neuroscientist looks neuroskeptically at neuroethics in the neuroworld**. In *The Neuroscientific Turn: Transdisciplinarity in the Age of the Brain*. Edited by Melissa M. Littlefield, Jenell M. Johnson. Ann Arbor: University of Michigan Press; 2012:199.

• Wolpe PR: **Neuroethics**. In *Encyclopedia of Bioethics*. 3rd edition. Edited by Stephen G Post. New York: Macmillan Reference USA; 2004:1894–1898.

## Conclusion: A “Participatory Bibliography”

This bibliography has been developed to address the prodigious growth, increased international publishing, and need for uniformity in the neuroethics’ literature. The included entries have been selected from numerous journal articles, books, book chapters, and digital documents, written in several languages. In this way, the present work is intended to provide a comprehensive, although not exhaustive, resource of available information on the topic. While the databases and online resources described are troves of neuroethics’ literature, it is important to bear a number of caveats to mind. First, the current trend of automated indexing can produce inaccurate retrievals by missing pertinent documents and/or by assigning inappropriate indexing terms to items. Second, coverage of journal titles can be truncated by faulty uploads. Third, in some cases the number of author-provided keywords can be limited due to space concerns.

Therefore, if you, the reader, find that an appropriate document is missing from this bibliography, please enter its citation in the online “Comment” section of this paper or email it to the bibliographic manager at: bioethics@georgetown.edu. Likewise, if any neuroethical programs/agendas have not been included in the listing, please provide this information for review, and accurately updated information will be included in a subsequent part of this series. It is our intent to develop this neuroethics bibliography as a “living document” that seeks to meet the needs of students, researchers, practitioners (of medicine, law, etc.), educators, and the public. As well, we invite readers to peruse similar repositories of neuroethics’ information, namely The Law and Neuroscience Bibliography maintained by the MacArthur Foundation Research Network on Law and Neuroscience (http://www.lawneuro.org/bibliography.php), and the Neuroethics Bibliography maintained by the Research Group on Neuroethics/Neurophilosophy at the Johannes Gutenberg University in Germany (https://teamweb.uni-mainz.de/fb05/Neuroethics/SitePages/Home.aspx) so as to engage the entries provided herein – and in subsequent parts of this series – as contributory to a broader and more-finely grained presentation of scholarly work in the field.

## Competing interests

The authors declare that they have no competing interests.

## Authors’ contributions

All authors contributed to study concept. LB and MD were responsible for data collection, interpretation, and manuscript preparation. JG was responsible for study design, data interpretation, and developing, revising and critical review of the manuscript. All authors were involved in, and have given final approval of the version of the manuscript to be published.
